# Editorial: Deception in Court—Open Issues and Detection Techniques

**DOI:** 10.3389/fpsyt.2020.00476

**Published:** 2020-05-25

**Authors:** Cristina Scarpazza, Giuseppe Sartori

**Affiliations:** Department of General Psychology, University of Padova, Padova, Italy

**Keywords:** lie detection, memory detection, forensic psychiatry, criminal cases, translational application

“Deceiving others. That is what the world calls a romance.”

– Oscar Wilde

Forensic psychiatric assessment is an extremely difficult task that is even more complicated by the risk of deception and malingering. Due to the high prevalence of the latter (around 40%) (1, 2), an accurate and thorough evaluation is a cornerstone issue in forensic practice. This is especially true in the case of insanity evaluation, where the assessment of psychiatric and cognitive symptoms is further complicated by the fact that these symptoms can be easily faked or exaggerated (3) for defensive purposes, although the majority of offenders found not guilty by reason of insanity have had previous contacts with psychiatric services. Taking this problem into consideration is even more important during evaluation of defendants who do not have had a previous psychiatric history.

The importance of assessing malingering is unfortunately still underestimated by clinicians, who usually are overconfident on their clinical ability to detect deceptive behavior (4). However, scientific research suggests that experienced individuals (i.e. judges, psychiatrists, etc) performance in detecting deception is only slightly better than chance (5). For these reasons, in the last few years, there has been increasing interest in the application of cutting edge methods for the detection of deception to enhance its accuracy in the legal setting.

The aim of this Research Topic is twofold: first, it aims to provide an updated overview of the techniques currently used to detect deception and malingering in court. Second, it aims to provide new perspectives, emerging concepts, and novel deception detection techniques that could potentially expand the future role of neuro-scientific evidences in court.

The Research Topic opens with a comprehensive review of approaches to detect malingering in forensic context (Walczyk et al.), where the authors summarize the strategies currently applied to detect malingering of psychiatric symptoms and cognitive impairment. Critically, the shortcomings of each method are described. The review also analyzes in detail behavioral, reaction based memory detection techniques, such as the Concealed Information Test (CIT), and the Autobiographic Implicit Association Test (aIAT). A final emphasis is placed on new methods, grounded on theoretical accounts of deception, attempting to induce greater cognitive load on liars than truth tellers.

Two original studies deepen our knowledge on classical lie detection techniques. First, the interesting work from Curci et al. investigates the accuracy of relying on experiential criteria as paraverbal aspects and cognitive complexity to identify liars from videotaped interview. The results confirm previous literature that the accuracy in discriminating liars from truth-tellers is far from accurate and that the identification of truth is more accurate than the one of lie. Critically, the study also highlights that judges' accuracy is poorly related to their confidence in their detection and this should be taken into account in real life settings to avoid wrongful decisions. Second, Mazza, Burla et al. used the Minnesota Multiphasic Personality Inventory-2-Restructured Form (MMPI-2-RF) in a very big sample (n=400) of post-divorce child custody court controversies, revealing that these individuals showed higher scores in underreporting and lower scores in overreporting validity scales. These results are critical as they highlight the necessity to interpret the MMPI-2 profile of these clients in light of normative data collected specifically in a forensic setting and the urgent need to identify alternative/complementary methods.

Five papers expanded the topic of CIT. Matsuda et al. shared their expert knowledge on the use of CIT in real criminal investigations in Japan, where the CIT is widely used in association with the polygraph to detect deception. Interestingly, they underline the difference between laboratory and field CIT and discussed some practical problems in its use and interpretation, such as the determination of statistical methods to be applied, the selection of a discriminative threshold to identify cheaters and the need to add additional measures to reduce the inconclusive cases. An original study by Ambach et al. focused on the impact of evaluative observation on psysiological responding in CIT. In a between-subjects manipulation, participants were divided into two groups, based on whether or not they were observed through a camera and were faced with the real-time video of the experimenter watching them while completing the CIT. Physiological measures were recorded. Results revealed that the expected enhanced CIT effect under evaluative observation was not present. A second study on CIT, by Rosenfeld et al., aims to investigate the influence of instruction and motivation on the P300 CIT effect and found that the financial motivation does not impact the P300 CIT effect and that financial incentives has no incremental effect after participants are instructed to defeat the test. The third original study of this section aims to differentiate between innocent suspects who have knowledge of crime information and guilty suspects. To this aim, Kim et al. used eye tracking to study eye movement of participants while viewing crime-relevant, crime-irrelevant and neutral stimuli. The interesting results revealed that guilty individuals show attentional avoidance as they focused their attention on crime-relevant and irrelevant stimuli for a shorter period of time compared with innocent individuals who have knowledge of crime. The potential translational application of these results is worth further investigations. The translational application of reaction-time (RT) CIT has been expanded by Suchotzki et al., where it has been used in a forensic sample submitted to an imaginary mock crime task. The data revealed that the RT CIT produced medium to large effects in both error rate and RTs, supporting the hypothesis that RT CIT is a promising techniques also in real life contexts. Second, the CIT effect was stronger in the inmate group compared to the control group, when error rates are analyzed. Third, the CIT effect does not correlate with impulsivity, rejecting the hypothesis that CIT effect in forensic samples can be attributable to differences in response inhibition.

Two interesting papers cover the topic of verbal lie detection and underline the need of more strategy-based research in the field of verbal lie detection. In particular, Vrij et al., besides providing a comprehensive review of the verbal lie detection techniques, focuses on the Model Statement, a technique where interviewers elicit participants to provide additional information on a specific topic. Based on prior knowledge of the different cognitive abilities used by individuals during truth telling or during lie, the method relies on the quality (rather than the quantity) of information that is reported to classify a narration as truthful or deceitful. Importantly, the authors describe how to use the model statement in real life, providing important and practical suggestions for scientists. The critical need to dig deeper into the language of liars, looking for traces of deception in the quality of the details provided during the narration is further expanded by Nahari and Nisin, who, in their opinion paper, wisely suggest to proceed with an in depth analysis of the narration, that, qualitatively, will greatly differ between truth tellers and liars.

Two papers cover the topic of detection of malingered amnesia for the crime. An interesting review by Jelicic summarizes the scientific evidences on crime related amnesia and described the methods actually used to evaluate its genuineness. Of particular relevance, the author also describe the approach to use the symptom validity testing strategy created on details from crime scenes and explains in which cases and why to adopt this approach can be considered more reliable than relying on other techniques to determine the authenticity of memory loss. The topic is further expanded by the original investigation from Zago et al., where the efficacy of three techniques, namely the aforementioned symptom validity testing, the facial thermography and the kinematic analysis of mouse movements, is compared with regard to the detection of feigned amnesia for crime. Besides confirming the efficacy of symptom validity testing in detecting feigned amnesia, the results also support the usefulness of kinematic analysis of mouse movements in differentiating truth tellers from liars in the case of amnesia malingering.

In the current Research Topic, new detecting deception techniques have also been proposed and their real potential translational application in court has been discussed. In particular, the fascinating idea of using the mouse trajectory dynamics as a tool for lie detection has been proposed in Monaro et al., where this technique has been used, during a two alternative forced choice task on symptoms of depression, to detect the simulation of depressive symptoms. The authors stressed that this tool has the key advantage that the kinematic movement is not consciously controllable by the individuals, and thus it is almost impossible to deceive. A complex data analysis performed using machine-learning models trained on mouse dynamics features, reached a classification up to 96% in distinguishing liars from depressed patients and truth-tellers. The usefulness of machine learning algorithms to enhance the accuracy detection of malingerers of psychopathology is also a key topic of Mazza, Monaro et al., where these algorithms applied to the Minnesota Multiphasic Personality Inventory-2 Restructured Form data, collected through a computerized form, revealed 95% of accuracy in detecting malingerers when subjects were instructed to respond under time pressure.

The Research Topic concludes with an important paper from Burgoon that, besides providing a summary of verbal and non verbal signals on which people rely to formulate gut judgments on authenticity, suggests to adopt an holistic approach based on the convergence of evidences principle, where multiple indicators of lie from different techniques are applied together to improve detection deception accuracy.

Lie and memory detection techniques are enormously promising as they have high potential translational value. As each method is characterized by specific drawbacks, scientists, and forensic experts should be well aware of them to select the most appropriate technique depending on their research or real life question, in order to enhance their future application into real world forensic practice. The innovative techniques discussed in this special issue are of interest both at the fact finding investigative stage (e.g. verbal lie detection) as well as in the verification stage (e.g. CIT). We hope that the readers will find this Research Topic a useful reference reflecting the current state of art in this emerging field of neuroscience based detecting deception tools.

## Author Contributions

CS and GS wrote the manuscript.

## Conflict of Interest

The authors declare that the research was conducted in the absence of any commercial or financial relationships that could be construed as a potential conflict of interest.
